# CD8^+^ T Cell Cross-Reactivity Profiles and HIV-1 Immune Escape towards an HLA-B35-Restricted Immunodominant Nef Epitope

**DOI:** 10.1371/journal.pone.0066152

**Published:** 2013-06-17

**Authors:** Chihiro Motozono, John J. Miles, Zafrul Hasan, Hiroyuki Gatanaga, Stanley C. Meribe, David A. Price, Shinichi Oka, Andrew K. Sewell, Takamasa Ueno

**Affiliations:** 1 Institute of Infection and Immunity, Cardiff University School of Medicine, Heath Park, Cardiff, United Kingdom; 2 Center for AIDS Research, Kumamoto University, Kumamoto, Japan; 3 Australian Centre for Vaccine Development, Human Immunity Laboratory, Queensland Institute of Medical Research, Brisbane, Australia; 4 School of Medicine, The University of Queensland, Brisbane, Australia; 5 AIDS Clinical Center, National Center for Global Health and Medicine, Tokyo, Japan; University of Alabama, United States of America

## Abstract

Antigen cross-reactivity is an inbuilt feature of the T cell compartment. However, little is known about the flexibility of T cell recognition in the context of genetically variable pathogens such as HIV-1. In this study, we used a combinatorial library containing 24 billion octamer peptides to characterize the cross-reactivity profiles of CD8^+^ T cells specific for the immunodominant HIV-1 subtype B Nef epitope VY8 (VPLRPMTY) presented by HLA-B^*^35∶01. In conjunction, we examined naturally occurring antigenic variations within the VY8 epitope. Sequence analysis of plasma viral RNA isolated from 336 HIV-1-infected individuals revealed variability at position (P) 3 and P8 of VY8; Phe at P8, but not Val at P3, was identified as an HLA-B^*^35∶01-associated polymorphism. VY8-specific T cells generated from several different HIV-1-infected patients showed unique and clonotype-dependent cross-reactivity footprints. Nonetheless, all T cells recognized both the index Leu and mutant Val at P3 equally well. In contrast, competitive titration assays revealed that the Tyr to Phe substitution at P8 reduced T cell recognition by 50–130 fold despite intact peptide binding to HLA-B^*^35∶01. These findings explain the preferential selection of Phe at the C-terminus of VY8 in HLA-B^*^35∶01^+^ individuals and demonstrate that HIV-1 can exploit the limitations of T cell recognition *in vivo*.

## Introduction

Hypervariable viruses such as HIV-1 can escape from human leukocyte antigen class I (HLA-I)-restricted CD8^+^ T cell responses by acquiring viral genomic mutations within or near immunogenic epitopes. Such immune escape pathways can be extremely reproducible and broadly predictable based on host HLA-I alleles at a population level [1], [2]. Somewhat paradoxically, however, antigen cross-reactivity is an inbuilt feature of the T cell compartment [3], [4]. Indeed, a single autoimmune T cell receptor (TCR) has recently been shown to recognize more than a million different peptides within a broad cross-reactivity profile encompassing unrelated amino acid substitutions [5]. Furthermore, several lines of evidence suggest that certain CD8^+^ T cell subsets with the capacity to cross-recognize naturally occurring viral variants are advantageous for viral control *in vivo* [6]–[11]. However, the true extent of HIV-1-specific T cell cross-reactivity remains elusive. In the present study, we characterized the cross-reactivity footprints of HIV-1-specific CD8^+^ T cells using combinatorial peptide library (CPL) scanning to cover all possible amino acid variations at each position of an octamer epitope. Additionally, we analyzed antigenic variation within the targeted epitope region of HIV-1 subtype B. Our investigations focused on CD8^+^ T cell responses specific for the immunodominant HIV-1 Nef epitope VY8 (VPLRPMTY) presented by HLA-B^*^35∶01 [12], [13].

## Materials and Methods

### Ethics Statement

All study participants provided informed, written consent at the AIDS Clinical Center, National Center for Global Health and Medicine, Japan. The study was approved by the Institutional Review Board of Kumamoto University and National Center for Global Health and Medicine.

### Sequence Analysis of Autologous HIV-1

Treatment-naïve individuals (n = 336) with chronic HIV-1 infection (>90% subtype B) attending the AIDS Clinical Center (International Medical Center of Japan) were enrolled for autologous HIV-1 sequence analysis. The median [IQR] plasma viral load was 95,000 [31,000–350,000] copies/ml; the median [IQR] CD4^+^ T cell count was 242 [64.5–367.5] cells/mm^3^. We determined autologous *nef* sequences from plasma viral RNA using a previously reported direct sequencing method [13].

### Generation and Maintenance of CD8^+^ T cell Lines and Clones

The CD8^+^ T cell clones (19–136, 19–139 and 33-S1) were established previously [13]. Additional CD8^+^ T cell lines and clones were generated by VY8 peptide stimulation of peripheral blood mononuclear cells (PBMCs) isolated from HLA-B*35∶01^+^ individuals with chronic HIV-1 infection (Pt-100 and Pt-168) with 10 nM of VY8 (VPLRPMTY) peptide. The Institutional Review Board of the National Center for Global Health and Medicine approved both taking samples and generating cell lines, and patients provided the written informed consent. All CD8^+^ T cell lines and clones were maintained in RPMI 1640 supplemented with 10% fetal calf serum, 10 IU recombinant human interleukin (IL)-2, antibiotics and L-glutamine.

### Analysis of TCR-encoding Genes

TCR-encoding genes of CD8^+^ T cell lines and clones were obtained by using a SMART PCR cDNA synthesis kit (Clontech) and analyzed with reference to the ImMunoGeneTics database (http://imgt.cines.fr) as described previously [14].

### T cell Sensitivity Assay

Secretion of cytokines and chemokines by virus-specific CD8^+^ T cells in response to specific antigen provides a useful tool for quantitative assessment of antigen recognition [15], [16]. MIP-1β was used as a functional readout in this study since it is one of the most sensitive means to assess functional avidity of human CD8^+^ T cells as previously described [15]–[17]. Briefly, 3×10^4^ T cells were mixed with 6×10^4^ HLA-B^*^35∶01-expressing C1R cells (C1R-B3501), either unpulsed or pulsed with cognate peptide across a range of concentrations. After overnight incubation at 37°C, the supernatant was harvested and assayed for MIP-1β content by ELISA as described previously [5], [17]. The amount of MIP-1β released in the absence of the peptide was subtracted as background. It should be noted that the VY8 peptide titration experiments of T cell clones 136 and 139 exhibited comparable results when IFN-γ [13] and MIP-1β were used as readouts (data not shown).

### Octamer Combinatorial Peptide Library (CPL) Scan

The octamer CPL contained a total of 2.4×10^10^ different peptides (PepScan) divided into 160 sub-mixtures in positional scanning format as described previously [4], [18]. Target C1R-B3501 cells (6×10^4^ cells/well) were pre-incubated in the absence or presence of CPL sub-mixtures (100 µg/ml). Effector T cells (3 x 10^4^ cells/well) were then added and incubated overnight at 37°C. Supernatant was collected and analyzed for MIP-1β content by ELISA as described previously [5], [17]. Background-subtracted results were expressed as % response, normalized with respect to the VY8 index residue. A response >20% was considered positive.

## Results and Discussion

### Clonotypic Characterization of VY8-specific T cells

CD8^+^ T cell lines were established from two HLA-B^*^35∶01^+^ individuals with chronic HIV-1 infection (Pt-100 and Pt-168). Analysis of TCR β usage by these T cell lines revealed multiple clonotypes, with 23 and 17 distinct TCR β sequences for Pt-100 and Pt-168, respectively (Table 1). This observation is consistent with previous studies showing the oligoclonal nature of immunodominant HIV-1-specific CD8^+^ T cell populations [19], [20]. The CD8^+^ T cell clones K51, K105 and K810 were generated from patient Pt-100 by limiting dilution of VY8-specific T cell lines. Monoclonality was confirmed by TCR β analysis and all three sequences were encompassed within the TCR repertoire of the parental T cell lines (Table 2). Additional CD8^+^ T cell clones (136, 139, and S1) previously established from two separate HLA-B^*^35∶01^+^ HIV-1-infected individuals [12], [13] showed distinct TCR β chain usage (Table 2) and were also used for cross-reactivity studies.

**Table 1:** TCR β composition of CD8^+^ T cell lines.

Patient	β chain			
	V gene	J gene	CDR3 sequence	Frequency
Pt-100	BV2*01	BJ2-7*01	CASSGEGNYEQYF	1/31
			CASTTDRVYEQYF	1/31
	BV3-1*01	BJ2-5*01	CASSTSSVTETQYF	2/31
		BJ2-7*01	CASSQDIAGVHEQYF	1/31
	BV4-1*01	BJ2-1*01	CASSQTSGSYNEQFF	1/31
	BV6-1*01	BJ1-5*01	CASSEASGIYEQYF	1/31
		BJ2-7*01	CASSEASGIYEQYF	1/31
	BV10-1*01	BJ2-1*01	CASSAAGVEYNEQFF	1/31
	BV11-2*01	BJ1-1*01	CASSFDIVNTEAFF	1/31
		BJ2-1*01	CASSPDLVDNEQFF	4/31
		BJ2-5*01	CASSGAWTGGGETQYF	2/31
		BJ2-7*01	CASSLDLVSYEQYF	1/31
			CASSLGIGRAYEQYF	1/31
	BV12-3*01	BJ1-4*01	CASSLRFATNEKLFF	1/31
	BV27*01	BJ2-5*01	CASSFDTNQETQYF	1/31
		BJ2-7*01	CASSLDTNGYEQYF	1/31
			CASSFQLAGVHGQYF	1/31
			CASSPRLDDEQYF	2/31
			CASSLDTSGYEQYF	2/31
			CASSSDREDSHEQYF	2/31
	BV28*01	BJ2-2*01	CASSSTDRAIPNTGELFF	1/31
		BJ2-3*01	CASSLPGLDSTDTQYF	1/31
		BJ2-7*01	CASSEGQGRYEQYF	1/31
Pt-168	BV2*01	BJ2-7*01	CASSESLAGGPYEQYF	7/31
	BV3-1*01	BJ2-3*01	CASSQEGADTQYF	2/31
	BV3-1*02	BJ2-3*01	CASSQEGAGTQYF	1/31
	BV6-2*01	BJ1-1*01	CASSGGRTDENTEAFF	1/31
		BJ2-1*01	CASSYEREDSGNEQFF	1/31
	BV11-2*01	BJ2-7*01	CASSLDVAGSYEQYF	1/31
			CASSLDIVSYEQYF	1/31
	BV11-3*03	BJ2-3*01	CASSLVLGTGTDTQYF	1/31
	BV12-3*01	BJ2-3*01	CASSWDSISTDTQYF	1/31
		BJ2-7*01	CASSSDGYEQYF	3/31
	BV12-5*01	BJ2-2*01	CASGLAMVVSGELFF	1/31
	BV15*02	BJ2-1*01	CATSRDLVEDEQFF	2/31
	BV20-1*05	BJ2-2*01	CSARDPRTDRGNTGELFF	1/31
	BV24-1*01	BJ2-3*01	CATSVRDDLTGNGPDTQYF	2/31
	BV27*01	BJ2-3*01	CASSLDLRPDTQYF	1/31
	BV28*01	BJ2-5*01	CASSLLGEETRETQYF	4/31
	BV30*01	BJ2-5*01	CAWHTVRVQETQYF	1/31

**Table 2:** TCR β composition of CD8^+^ T cell clones.

Patient	Clone	β chain		
		V gene	J gene	CDR3 sequence
Pt-19	19-136	BV7-2*03	BJ2-1*01	CASSPTPQGDYEQFF
				
	19-139	BV11-2*01	BJ1-1*01	CASSLDLVSTEAFF
				
Pt-33	33-S1	BV4-2*01	BJ2-3*01	CASSQAADAAITDADTQYF
				
Pt-100	100-K51	BV27*01	BJ2-5*01	CASSFDTNQETQYF
				
	100-K105	BV11-2*01	BJ1-1*01	CASSFDIVNTEAFF
				
	100-K810	BV27*01	BJ2-7*01	CASSFQLAGVHGQYF

### Cross-reactivity Analysis of VY8-specific T cells

**Figure 1:**
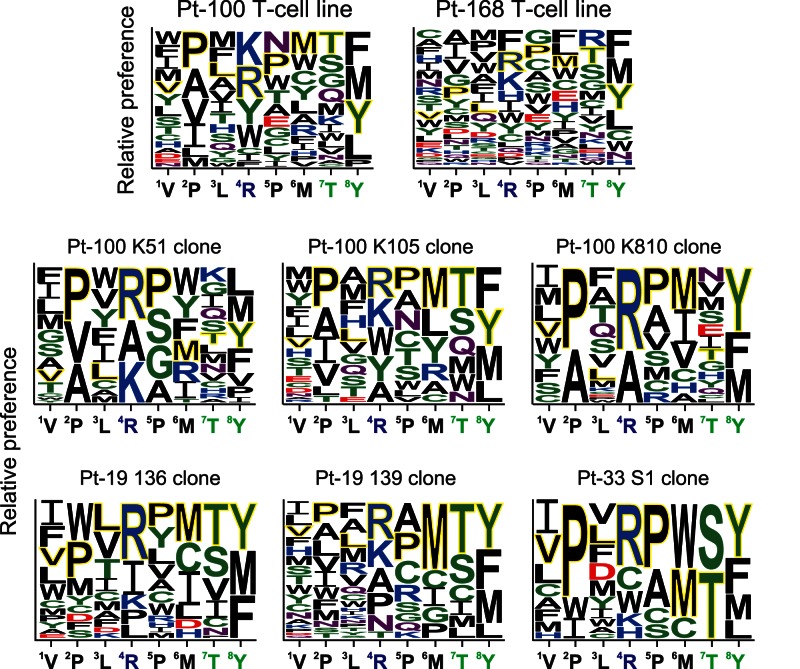
Amino acid residues preferentially recognized by VY8-specific CD8^+^ T cells. Graphical representation showing relative preference for amino acid residues recognized by VY8-specific T cell lines and clones based on the CPL scan data shown in Figure S1. Responses >20% were included. A web-based application, WebLogo 3 (http://weblogo.threeplusone.com/), was used to generate the graphic. Colours represent physicochemical properties: polar (G, S, T, Y and C), green; neutral (Q and N), purple; basic (K, R and H), blue; acidic (D and E), red; hydrophobic (A, V, L, I, P, W, F and M), black. The index residues at each position are outlined in yellow. Residue size is proportional to T cell recognition preference.

The cross-reactivity profiles of VY8-specific T cell lines and clones were analyzed using a CPL containing a total of 2.4×10^10^ different octamer peptides, which allowed qualitative mapping of preferred T cell recognition residues at each position along the peptide backbone [4], [18]. Different VY8-specific T cell lines and clones preferentially recognized different amino acid residues across the octamer peptide backbone (Figure S1). We employed a graphical representation of these preferential recognition residues by the VY8-specific T cells (Figure 1). Despite these unique cross-reactivity patterns, all T cells tested recognized the index VY8 residues efficiently (Figure 1). This finding contrasts with previous observations using tumor-specific and autoreactive T cell clones [5], [21]–[23], which typically prefer non-index amino acid residues. Across all clones, more stringent recognition was observed at position 2 (P2) and P8 (Figure 1). This most likely reflects the anchor role of these positions in peptide binding to HLA-B^*^35∶01 [12], [24]. The VY8-specific T cell clones, K51, K105 and K810, showed inherently unique cross-reactivity footprints but less flexible cross-recognition compared to the parental T cell line (Figure 1), suggesting increased coverage of viral antigenic variation through polyclonal TCR cross-reactivity.

### Naturally Occurring Antigenic variations within the VY8 Epitope

**Figure 2:**
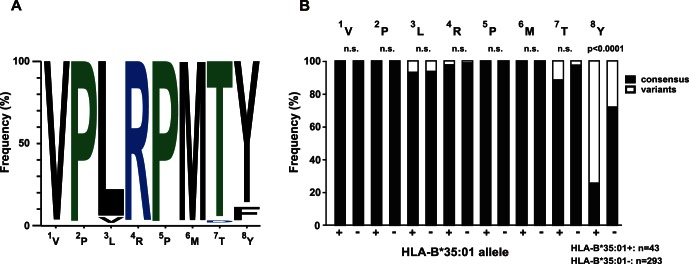
Naturally arising antigenic variations in the VY8 epitope. (**A**) Graphical representation showing the frequency of amino acid residues within the VY8 epitope in subtype B Nef sequences retrieved from the Los Alamos database (n = 1191). WebLogo 3 was used to generate the graphic. (**B**) The frequency of consensus (subtype B) and variant amino acid residues at each position of the VY8 epitope is shown for autologous plasma viral sequences derived from a total of 336 HIV-1-infected individuals, segregated according to HLA-B^*^35∶01 status. Statistical analysis was performed using Fisher’s exact test. *n.s*., not significant.

To investigate the correlation between T cell cross-reactivity and naturally occurring antigenic variation, we analyzed sequence polymorphisms within the VY8 epitope. Despite the remarkable variability of HIV-1 Nef, VY8 is highly conserved, most likely due to its location partially within a Src homology 3 binding motif that is critical for several Nef functions [25], including HLA-I down-regulation [13], [26]. Nevertheless, in the Los Alamos HIV Sequence database (http://www.hiv.lanl.gov/content/index), some variability within HIV-1 subtype B has been reported at P3 Leu and P8 Tyr of the VY8 epitope, with 2.4% and 8.2% of viral clones showing polymorphisms in these positions, respectively (Figure 2A). Given that approximately 40% of Nef sequence polymorphisms are associated with host HLA-I alleles [1], we examined these particular variants for HLA-I association. Our previous smaller study of 69 HIV-1-infected patients indicated that Phe at P8 might be associated with the HLA-B^*^35∶01 allele [13]. To confirm this association and examine polymorphisms at P3, we recruited a larger cohort comprising 336 treatment-naïve individuals with chronic HIV-1 infection and determined autologous *nef* sequences from plasma viral RNA. Although we found some variability at P3 (3%), there were no statistically significant amino acid differences at P1–P7 between individuals with or without HLA-B^*^35∶01 (Figure 2B). In fact, CPL scanning showed that, at P3, hydrophobic residues including both the index Leu and mutant Val were preferentially recognized by all VY8-specific T cells tested (Figure 1). Such flexible TCR recognition at P3 helps to explain why the Val mutant is not selected in HLA-B^*^35∶01^+^ individuals. Conversely, we found a statistically significant difference in the frequency of polymorphisms at P8 between individuals with or without HLA-B^*^35∶01 (Figure 2B); indeed, the vast majority (74%) of HLA-B^*^35∶01^+^ donors harboured viral sequences with Phe at P8. However, CPL scanning showed that Phe was a favoured amino acid residue recognized by T cell lines and some clones, such as K105 (Figure 1 and Figure S1). In these instances, CPL data alone do not simply explain the emergence of this viral mutation in HLA-B^*^35∶01^+^ individuals.

### VY8-specific T cell Sensitivity Towards Peptide Variants

**Figure 3:**
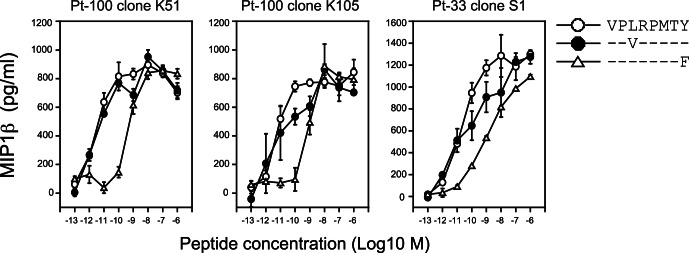
VY8-specific CD8^+^ T cell sensitivity towards peptide variants. The sensitivity of T cell clones towards the VY8, VY8-3V and VY8-8F peptides was quantified by measuring the amount of MIP-1β secreted in response to antigen stimulation. Data are representative of duplicate assays and standard deviation from the mean of two replicates is shown.

To verify the effect of single mutations within the VY8 peptide on TCR sensitivity, we performed competitive titration assays across our panel of VY8-specific T cells (Figure 3). Consistent with the CPL scan data, all T cells tested recognized the VY8 and VY8-3V peptides comparably (<2 fold difference in EC_50_ values; Table 3). In contrast, the EC_50_ values for VY8-8F were >50 fold higher than index for all T cells tested (Table 3). These observations are consistent with previous reports showing that VY8-specific T cells could not recognize CD4^+^ T cells or macrophages infected with HIV-1 carrying this Nef variant at P8 [13], [26].

**Table 3:** Sensitivity of VY8-specific CD8^+^ T cells.

CD8^+^ T cells		EC_50_ (M)		
		VY8	VY8-3V	VY8-8F
lines	Pt-100	5.9×10^−12^ (x 1)	nd	3.9×10^−10^ (x 66)
	Pt-168	4.0×10^−12^ (x 1)	nd	4.3×10^−10^ (x 105)
clones	33-S1	2.3×10^−11^ (x 1)	3.9×10^−12^ (x 0.17)	1.2×10^−9^ (x 52)
	100-K51	3.1×10^−12^ (x 1)	5.8×10^−12^ (x 1.8)	4.2×10^−10^ (x 135)
	100-K105	5.1×10^−12^ (x 1)	3.9×10^−12^ (x 0.76)	6.7×10^−10^ (x 131)

EC_50_, determined by duplicate assays; nd, not done; in parenthesis, fold changes in sensitivity relative to index.

Although P8 is an anchor residue for VY8, our previous HLA-I stabilization studies showed comparable binding activity between HLA-B^*^35∶01 and either VY8 or VY8-8F [13]. The crystal structure of the VY8/HLA-B^*^35∶01 complex shows that P8 Tyr lies deep inside the F pocket of the HLA-I molecule [24]. Substitution at this position with the aromatic residue Phe may not induce substantial structural changes. Consequently, impaired T cell recognition of P8 Phe may be mediated by indirect conformational changes imposed by the peptide upon TCR binding [17]. In the context of HLA-A^*^02∶01, however, a Tyr to Phe substitution at the secondary anchor P3 of an antigenic peptide (SLFNTVATL) leads to unexpectedly large conformational changes in the peptide backbone [27]. Accordingly, further structural studies are needed to elucidate the precise mechanism through which anchor residue substitution leads to impaired T cell recognition of the VY8 epitope.

Previous studies have shown that the double substitution of Arg-71 to Thr and Tyr-81 to Phe (P8 at VY8) [13], or Pro-75 to Ala (P2 at VY8) as a single mutation, impair Nef-mediated down-regulation of HLA-I and thereby increase the susceptibility of HIV-1-infected cells to killing by CD8^+^ T cells targeting other epitopes [26], [28]. In contrast, the Tyr-81 to Phe (P8 at VY8) mutation alone exerts virtually no effect on Nef-mediated activities [13], [26]. Collectively, these data suggest that the P8 Phe mutation does not compromise viral fitness.

### Concluding Remarks

CD8^+^ T cell responses against the immunominant HIV-1 subtype B-derived Nef epitope VY8 presented by HLA-B^*^35∶01 are highly polyclonal, broadly cross-reactive and capable of tolerating natural viral variation with one notable exception. Specifically, the observed Phe substitution at P8, which is neutral in terms of Nef-mediated function [13], [26], was found to reduce CD8^+^ T cell recognition by >50 fold. The association of this mutation with HLA-B^*^35∶01^+^ strongly suggests that evasion of VY8-specific CD8^+^ T cell activity confers a selection advantage *in vivo*. Thus, even CD8^+^ T cell responses with extensive cross-reactivity profiles can succumb to immune escape at a single position.

## Supporting Information

Figure S1 CPL scanning of VY8-specific CD8^+^ T cells. The cross-reactivity profiles of T cell lines and clones specific for VY8 were tested by using 160 CPL sub-mixtures (100 µg/ml) comprising a total of 2.4×10^10^ different octamer peptides. In every peptide mixture, one position has a fixed amino acid residue and all other positions are degenerate, with the possibility of any one of 19 natural amino acids being incorporated in each individual position (cysteine is excluded). The amount of MIP-1β secreted in response to antigen was quantified by ELISA. Data are background-subtracted and the relative T cell response is shown as a ratio of MIP-1β production with respect to the index residue at each position. Responses >20% were considered positive and used to construct Figure 1. A representative set of duplicate assays is shown. Red bars depict residues corresponding to the VY8 index sequence.
(EPS)
